# Circular depolarization spectroscopy: A new tool to study photo‐imprinting of chirality

**DOI:** 10.1002/chir.23527

**Published:** 2023-01-13

**Authors:** Chidambar Kulkarni, Hirotoshi Sakaino, Ghislaine Vantomme, Stefan C. J. Meskers

**Affiliations:** ^1^ Institute for Complex Molecular Systems and Molecular Materials and Nanosystems Eindhoven University of Technology Eindhoven Netherlands; ^2^ Department of Chemistry Indian Institute of Technology (IIT) Bombay Mumbai India; ^3^ Electronic & Imaging Materials Research Laboratories Toray Industries, Inc. Otsu Shiga Japan

**Keywords:** azobenzene photo‐isomerization, cholesteric polymers, circular polarization, kinetic Monte Carlo simulations, photon counting, *π*‐conjugated polymers

## Abstract

When irradiating a molecular material containing photo‐isomerizable groups with pure circularly polarized light, a particular handedness may get imprinted into the material. To study the mechanism and kinetics of this process *in situ* and *operando*, we have developed a new chiroptical tool where the circular polarization of the incident circularly polarized light is monitored after transmission through the photoactive layer. Practical limits to the resolution and sensitivity of the measurements as well as its calibration are discussed. To aid interpretation of experimental results, we present kinetic Monte Carlo simulations on a model for the active material involving photo‐induced reorientation of molecules in a cholesteric organization. The simulations support the interpretation of a transient minimum in the degree of circular polarization of the transmitted light in terms of a nematic transient state during photo‐inversion of a cholesteric organization in the molecular material.

## Graphical abstract

Circular depolarization is a new spectrosopic tool to study photo‐imprinting of chirality in films of photo‐isomerizable polymer in situ and *operando*.

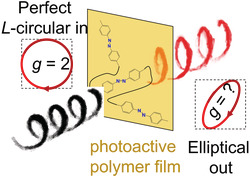

## 1 INTRODUCTION

Irradiation of racemic or achiral molecules and materials with circularly polarized light of a particular handedness can result in a breaking of the mirror‐image related symmetry and produce molecules with a net enantiomeric excess or a material with a helical organization.^1^ Various molecules, materials, and mechanisms have been studied. Interestingly, the process has also been proposed as a possible explanation for the homochirality found in nature.^2^

In case of a racemic mixture of chiral molecules dispersed in solution, circularly polarized light can be used to preferentially consume one of the enantiomers via an irreversible photochemical reaction.^3^ By judicious use of amplification associated with crystallization of the desired product and racemization of the unwanted isomer, the chemical yield of the desired enantiomer can be dramatically improved.^4^ Circularly polarized light can also be used to induce chirality in inorganic nanoparticles^5^, ^6^ and polymers.^7^, ^8^, ^9^

Illumination with circularly polarized light can be very effective in inducing helical order in supramolecular aggregates^10^ and liquid crystalline (LC) phases.^11^, ^12^, ^13^ This effectiveness becomes understandable if one considers that systems whose helical internal organization is characterized by a pitch length comparable to the wavelength of light show a particularly strong chiral discrimination in their interaction with chiral photons (circularly polarized light),^14^ namely, the circular selective reflection by cholesteric liquid crystals.

Recently, we have shown that circularly polarized light can be used to photo‐imprint chirality in films of π‐conjugated polymers with an azobenzene moieties in the main chain.^15^, ^16^, ^17^, ^18^ The possibility to reversibly impose a particular organization on a polymer film opens opportunities to make optical elements to control the polarization state of optical beams. Beams with orbital angular momentum are of particular interest.^19^, ^20^ Furthermore, materials with externally controllable helicity may be useful in molecular separations^21^ and perhaps as spin filter for electrons.^22^ Finally, if the photoactive material bears stable, enantiopure stereo centers, there is the possibility that the photo‐irradiation in combination with chiral friction^23^ gives rise to unidirectional rotation.^24^

Our mechanistic understanding of the photo‐imprinting of chirality in macromolecular materials is still only rudimentary.

In order to monitor the changes and better understand the mechanism involved, *in situ* and *operando* spectroscopic tools would be extremely useful.^25^, ^26^ With this aim, we have developed a new chiroptical technique, dubbed circular depolarization (CDP).

**FIGURE 1:**
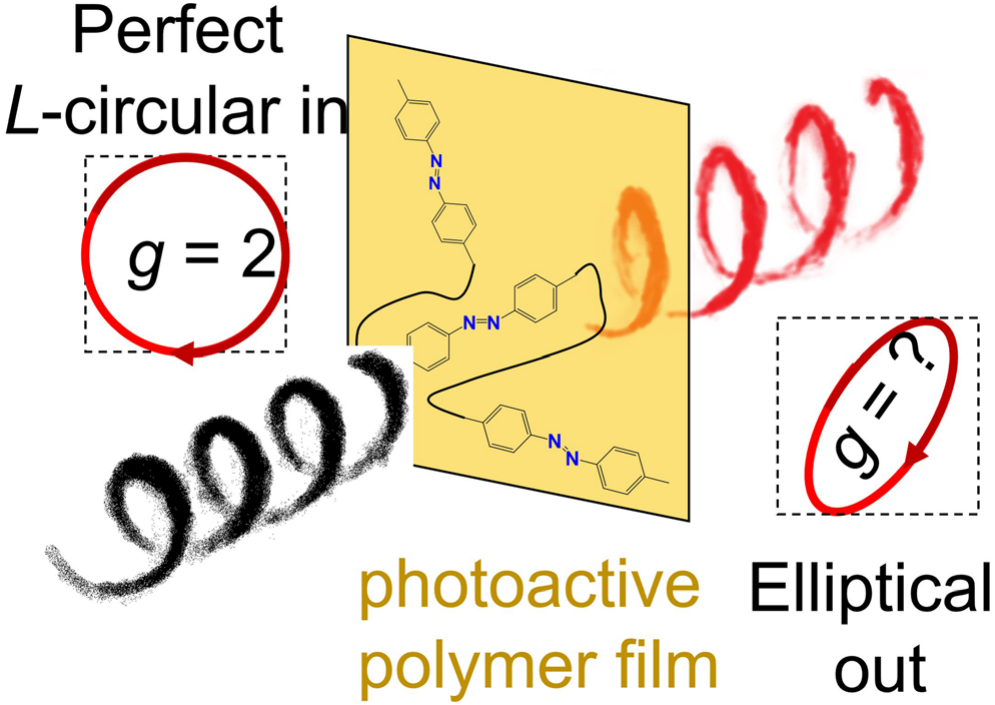
The principle of circular depolarization measurements on films of photoactive polymer

The principle of the CDP measurement is illustrated in Figure 1. Purely circular polarized light with, say, left‐handed polarization, is sent through a thin film of polymer containing photoactive azobenzene units. The wavelength of the incident light is such that it can be absorbed by the molecules. In the materials studied so far, the azobenzene moieties are part of the larger π‐conjugated electron system, and selective excitation of either cisoid or transoid azobenzene segments is unlikely. Nevertheless, in general, photo‐excitation of the polymer will result in random motion of the polymer. Then, if the new orientation of the polymer is such that the probability for light absorption is small, the chain will be kinetically trapped and remain in its particular orientation for a long time.

At the end of the imprinting experiment, the photo‐induced organization of the polymers is fixated by lowering the temperature or removing plasticizing solvent by evaporation. The final vitrified molecular order is probed by circular dichroism spectroscopy. Although this procedure to find light‐induced helicity works well, it requires many separate runs to probe the temporal evolution of the internal structure during illumination. An *in situ* probe can significantly shorten the time needed for experimentation and provide mechanistic insights on the evolution of helical structure.

**FIGURE 2:**
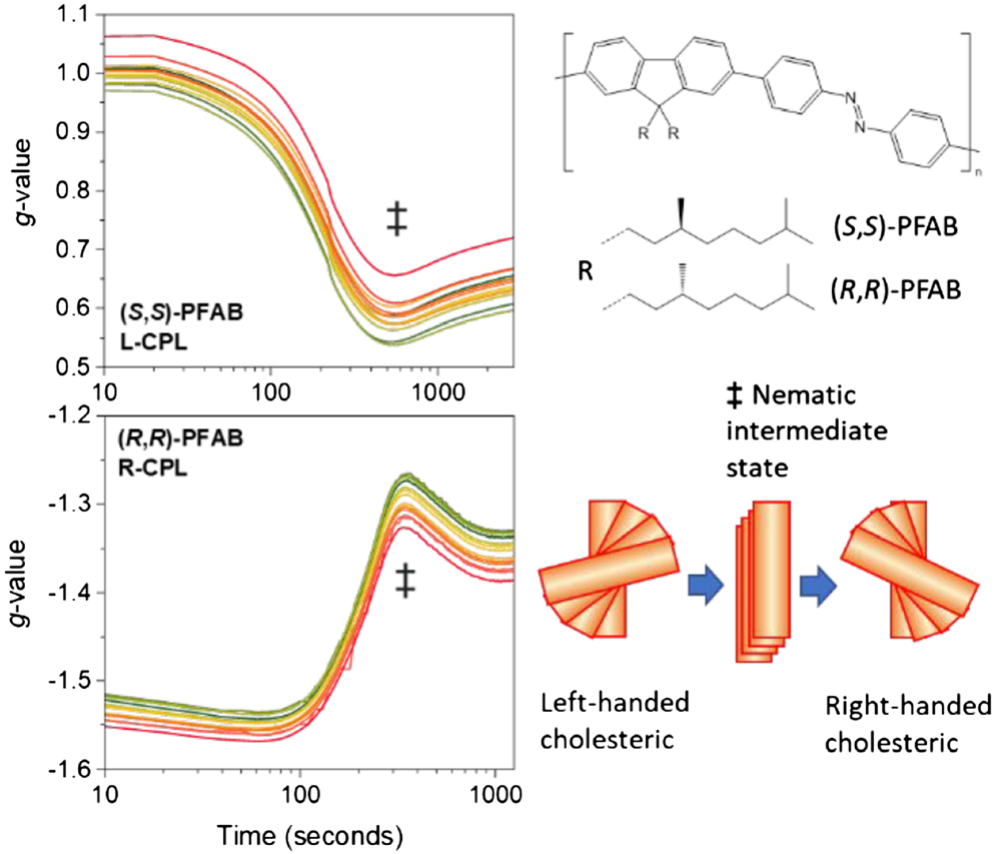
Circular depolarization measurements on thermally annealed films of (*S*,*S*)‐PFAB polymer with pure left circularly polarized light (top panel) and (*R*,*R*)‐PFAB polymer with pure right circularly polarized light (bottom panel) of 455 ± 10 nm wavelength from an LED. The vertical axes show the dissymmetry ratio for the light after transmission through the film. The lines show measurements at different wavelengths separated by 0.9 nm, with the green line corresponding to the shortest wavelength. The right side shows the structure of the polymer and its two enantiomeric forms. The feature in the kinetic traces labeled ‡ is interpreted as the transient nematic intermediate state of the light induced inversion of the cholesteric helical arrangement. Film thickness: 130 nm, max. OD ~ 1

Any order in the polymer film will affect the polarization of light as it travels through a film. Hence, the light that is transmitted in an imprint experiment will likely have a polarization that is no longer purely circular but, in general, elliptical. By analyzing the degree of circular polarization of this transmitted light in real time during the irradiation, one can monitor the kinetics of the changes induced in the film by the circularly polarized incident light. An example of a CDP measurement is illustrated in Figure 2.

In this contribution, we describe the CDP measurement technique and discuss accuracy, sensitivity, and time resolution. Beside instrumental tools, also, theoretical methods are needed to interpret results. In the second half of this contribution, we detail a possible microscopic model for the photo‐imprinting. Using this model and the kinetic Monte Carlo method, we then simulate a CDP transient. This then allows us to assign intermediate features in the experimental CDP results illustrated in Figure 2.

## 2 MATERIALS AND METHODS

### 2.1 Detection scheme for circular depolarization

**FIGURE 3:**
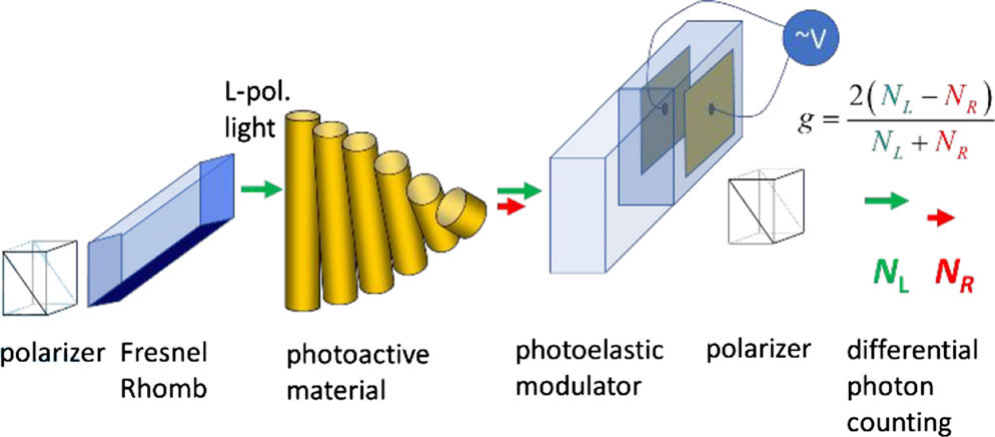
Technical details of the circular depolarization measurement using the differential photon counting scheme

The spectroscopic setup used for the CDP measurements is illustrated in Figure 3. Here, light from an intense source (e.g., an LED) is first sent through a linear polarizer and subsequently through a Fresnel rhomb (see Supporting Information). This results in highly circularly polarized light with dissymmetry ratio *g* = 2(*I*_L_−*I*_R_)/(*I*_L_ + *I*_R_) that typically exceeds 1.85 in absolute magnitude. In the expression for *g*, *I*_L_ (*I*_R_) denotes the intensity of left (right) circularly polarized light. The advantage of the Fresnel rhomb when circularly polarizing the light is that a high degree of circular polarization can be achieved over a broad spectral range, so that one is not restricted to quasi‐monochromatic light sources. The circularly polarized light is sent through the photoactive polymer film. The residual beam that is transmitted through the film is then sent through a photoelastic modulator (Hinds, PEM 90). The modulator, in combination with a linear polarizer directly behind it, alternatingly lets through left and right polarized light and operates at high frequency (50 kHz). The transmitted beam is then focused onto a slit and sent through a spectrograph to separate different wavelength components and reject unwanted photoluminescence from the film. The spectrally dispersed light is sent to a photomultiplier array (Hamamatsu, RG 5900U‐03‐L16) that operates in photon counting mode. This allows us to monitor the photon rates of 16 different wavelength components simultaneously. The photocurrent pulses are digitized and separated according to the circular polarization of the original photon using electronic time gates and then counted. This differential photon counting (DPC)^27^, ^28^ yields numbers *N*_L_ and *N*_R_ for the total count of left and right polarized photons. With these, the degree of polarization of the light is computed *g* = 2(*N*_L_−*N*_R_)/(*N*_L_ + *N*_R_). The main advantage of this method is that the time gates can be adjusted such that photons are counted only during parts of the modulation cycle of the photoelastic modulator where the modulation depth is sufficiently deep. This procedure minimizes contribution for linearly polarized light and yields absolute degrees of polarization, obviating the need for external calibration.^29^ The DPC method yields a stable baseline for *g* that differs less than 10^−5^ from zero, as per experiments using unpolarized light. Important in the context of the high circular polarization of light in the CDP experiment is that the DPC method operates reliably for *g* values spanning the entire range of possible values from −2 via 0 to +2.

Because depolarization is a very widespread optical phenomenon, the method is in principle also applicable to materials with a higher degree of structural order, although interpretation of the results may be more difficult. We note that besides the *g* value, also, the total transmitted is an observable. The latter quantity provides some information on induction of alignment of the chromophores in the direction perpendicular to the surface of the film. Furthermore, because the irradiating beam is passed through a monochromator, the method is also amenable to fluorescent materials as the relatively weak fluorescence can easily be filtered out.

The digital photon counting method has a limitation at high photon fluxes. Below, we analyze this limit and investigate systematic errors and sensitivity limits for single run photo‐isomerization experiments. In photon counting detection, the digitization inadvertently introduces a dead time after recording and detecting a photon pulse, during which no additional photon pulses can be registered. This is often referred to as pulse pile‐up. This effect results in a systematic error in the *g* values from DPC at high count rates.

From probability theory, we know that the chance for the occurrence of *n* events in a particular time interval, given the average number *λ* of such events per interval, can be calculated from the Poisson distribution *P*(*n*,*λ*). The probability *I*_count_ for registering an event in a period equal to the dead time of the detection system Δ*τ* is the given by

(1) $$I_{\text{count}}\left(\lambda \right)=\sum_{n=1}^{\infty }P\left(n,\lambda \right)=\sum_{n=1}^{\infty }\frac{\lambda^{n}}{n!}e^{-\lambda }=1-e^{-\lambda }.$$

**FIGURE 4:**
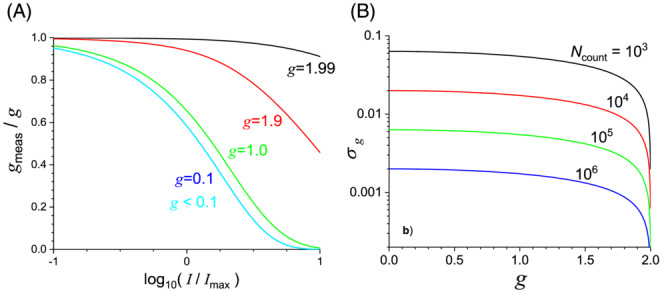
(A) Ratio of measured and real degree of circular polarization in an idealized differential photon counting detection system as function of the ratio between incident photon rate *I* and the maximum count rate of the detection system *I*_max_ for various values of the true degree of circular polarization *g* of incoming light. (B) Estimated standard error for the degree of polarization *g* as function of *g* and parametric in the total number of photons counted in the experiment determining *g*

For simplicity, we assume that at very low photon fluxes, the detector has unit detection efficiency. A detection system based on DPC thus returns a value for the dissymmetry ratio equal to

(2) $$g_{\text{meas}}\left(\lambda_{L},\lambda_{R}\right)=2\frac{\left(1-e^{-\lambda_{L}}\right)-\left(1-e^{-\lambda_{R}}\right)}{\left(1-e^{-\lambda_{L}}\right)+\left(1-e^{-\lambda_{R}}\right)}=2\frac{\left(e^{-\lambda_{R}}-e^{-\lambda_{L}}\right)}{\left(2-e^{-\lambda_{L}}-e^{-\lambda_{R}}\right)},$$

where *λ*_L(R)_ stands for average number of left (right) polarized photons arriving at the detector in an interval of length Δ*τ*. Using the elementary relations *λ*_L_ = *λ*_avg_ (2 + *g*)/2 and *λ*_R_ = *λ*_avg_ (2‐*g*)/2, we can then relate the measured degree of polarization *g*_meas_ and the true *g*. The ratio of measured and real *g* is shown in Figure 4A as function of the incident intensity *I* relative to the maximum count rate of the detection system *I*_max_ = 1/Δ*τ*.

As can be seen in Figure 4A, for a very high degree of circular polarization close to the maximum of 2, there is little systematic error even at very high intensity where severe pulse pile‐up occurs. For small *g* values of ≤0.1, however, severe deviations of the measured from the true value occur at incident photon rates approaching and exceeding the maximum count rate of the system. For modern detectors, dead times are shorter than 100 ns, and correspondingly, maximum count rates exceed 10^7^ per second. Then, upon accepting systematic errors up to 5%, one can use count rates up to 10^6^ Hz.

Having established now the maximum count rate, Poisson statistics also provides us with an estimate for the standard error of measurement. Following the standard rules for calculating the variance of *g* in terms of the number of left and right photons counted, one obtains the following expression of the standard deviation of *g* as function of the total number of photons counted *N*_tot_ and the degree of polarization *g*:

(3) $$\;\sigma_{g}\left(N_{\mathrm{tot}},g\right)=\sqrt{\frac{4-g^{2}}{N_{\mathrm{tot}}}}.$$

Relation (3) is illustrated in Figure 4B. Taking now the optimal count rate of 10^6^ Hz derived above, it follows from Figure 4B that standard errors of 10^−3^ are expected. Thus, we conclude that using the photon counting detection scheme and adjusting the transmitted intensity to 10^6^ per second, the circular depolarization of transmitted light should be measurable with 1 s time resolution, >95% accuracy, and 0.001 precision. This is consistent with experiment as shown in Figure 1.

### 2.2 Optical model and kinetic Monte Carlo simulation of photoimprinting of chirality

So far, materials for which photo‐imprinting of chirality and CDP monitoring have been successful have mainly been of the (chiral) nematic liquid crystal type. Chiroptical properties of such materials can be described by an optical model involving birefringent layers in a helical arrangement and the associated Maugin‐Oseen‐deVries^30^, ^31^ theory. Here, we follow this approach. First, we present a simple bilayer optical model that may account for photo‐imprinting of chirality. Later, we will extend the model to multiple layers and demonstrate simulation of circular depolarization.

**FIGURE 5:**
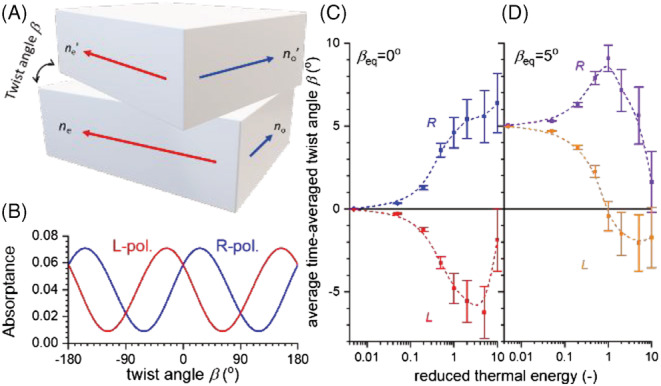
(A) Two‐layer optical model for chiral imprinting with variable twist angle *β* between the optically anisotropic layers with *ñ*_e_ = 1.8–0.4*i*, *ñ*_o_ = *n*_o_ = 1.2. (B) Absorptance for left and right circularly polarized light for the two layer model shown in (A) as function of the twist angle *β.* (C) Average twist angle induced by irradiation with left and right circularly polarized light in the two layer model system with nematic order under thermal agitation as function of reduced temperature. The average is calculated by taking the time average twist angle from 500 different kinetic Monte Carlo simulation runs. The error bars indicate the uncertainly of the average. (D) Same as (C) by now for a system showing cholesteric order number of isomerization steps in each run 1.2 × 10^4^

Figure 5A schematically depicts two identical layers of birefringent optical material stacked on top of each other with a twist angle *β*. The in‐plane optical anisotropy of both layers is described by refractive indices *n*_e_ and *n*_o_. A non‐zero twist angle between axes of the two layers makes the system chiral. Using elementary optics^32^ and introducing complex refractive indices *ñ*_e_ and *ñ*_o_, we can calculate the fraction of incident energy absorbed in a narrow zone around the interface between the two layers (5 nm) of a light beam incident along the normal. The fraction of light absorbed (=absorptance) is calculated for both left and right polarized incident light, and these two absorptances are shown in Figure 5B as function of the twist angle. As expected for a chiral system, there is a difference in absorptance for left and right circularly polarized light for twist angle *β* ≠ 0. We then assume that each photon absorbed in the interface region triggers an isomerization event in the molecular material resulting in a change of the twist angle *β*. The rates for a clockwise or anticlockwise change from initial twist angle *β* to *β* ± Δα are taken to be proportional, respectively:

(4) $$\begin{array}{l} k_{+}\left(\beta ,\Delta \alpha \right)\propto e^{\cos\left(2\left(\beta +\Delta \alpha -\beta_{\mathrm{eq}}\right)\right)/k_{b}T},\\ k_{-}\left(\beta ,\Delta \alpha \right)\propto e^{\cos\left(2\left(\beta -\Delta \alpha -\beta_{\mathrm{eq}}\right)\right)/k_{b}T}. \end{array}$$

In these rates, *β*_eq_ denotes an equilibrium twist angle, which the system adopts as average twist angle after random thermal agitation. *β*_eq_ = 0 corresponds to nematic ordering of the layers, while *β*_eq_ ≠ 0 indicates a cholesteric arrangement. *k*_*b*_ *T* denotes a reduced thermal energy expressed in units of the half of the maximal energy barrier for rotation. To simulate dynamic processes induced by, say, left circularly polarized light, we use the kinetic Monte Carlo (kMC) method. First, the absorptance for *L* photons is calculated for the current value of *β*. This probability together with a random number from a random number generator is used to set the time step for the next event. Then, a second random number is used to take the magnitude of the change in twist angle (Δ*α*) from a uniform distribution between 0 and 1. Finally, a third random number is used to select clockwise or anticlockwise rotation based on the different average rates for these two directions as expressed in Equation (4). These simulation steps are then iterated to get a run. After terminating a run, a time‐averaged value for *β* is calculated. By doing many runs, we obtain an average, time‐averaged value for *β* as well as a standard error *σ* for this average. Results for the average and its error are shown in Figures 5C,D. Figure 5C pertains to *β*_eq_ = 0° with blue and red symbols representing results for irradiations with *R* and *L* circularly polarized light. The average angles are plotted versus the reduced thermal energy. As can be seen, when the temperature is low the system shows nematic order, but when the thermal energy becomes comparable to the rotational energy barrier, left and right polarized irradiations induce opposite twist angles, consistent with photo‐imprinting of chirality. With increasing temperature, the standard error for the average also increases. The simulation reproduces the experimental finding that for successful photo‐imprinting of chirality, the chains need to be allowed sufficient freedom of movement by plasticizing the material with solvent vapor or choosing a temperature close to the nematic‐isotropic phase transition.

Using the kinetic Monte Carlo method, we can also simulate a system with preference for cholesteric order by introducing a non‐zero value for *β*_eq_ in Equation (4). Results are shown in Figure 5D. At low temperature, the chiral nematic order prevails, yet at thermal energies comparable to the rotation barrier, circularly polarized light is predicted to overrule the influence of chiral molecular structure, resulting in oppositely signed twist angles for left and right polarized irradiation.^13^ Note that for high temperatures, the average twist angles under left and right polarized irradiation do not have the same absolute magnitude, underscoring the diastereomeric relation between the two separate irradiation experiments with *L* and *R* light on the intrinsically chiral bilayer.

### 2.3 Helix inversion monitored via circular depolarization

**FIGURE 6:**
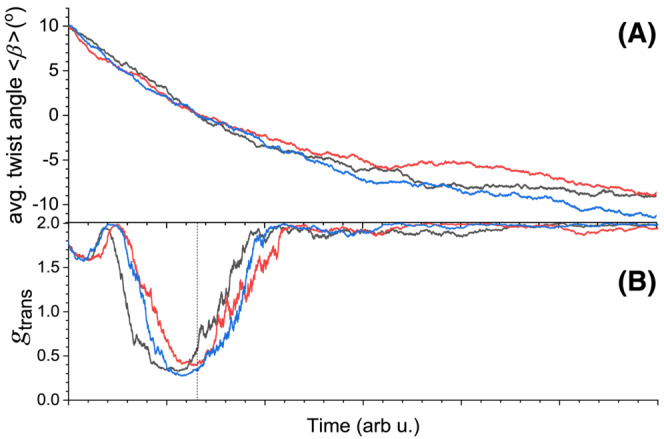
Kinetic Monte Carlo simulation of a thermal helix inversion in a stack of 30 layers for anisotropic material as shown in Figure 5. (A) Average twist angle of all 30 layers as function of time in a single simulation run with 10^4^ steps. Probabilities for isomerization events are based on Equation (4) with *β*_eq_ = −10°. At the start, all *β*'s are set to +10°. The three traces shown represent three different simulation runs with identical settings. (B) Degree of circular polarization of the light transmitted (*g*_trans_) through the stack of 30 layers during helix inversion

We now turn to a simulation of the helix inversion experiment shown in Figure 1. For a realistic representation of the optical properties of the polymer film in the simulation, we consider a stack of 30 dichroic layers as shown in Figure 5A. The thickness of the film is set to 200 nm. Yang and Mi^32^ have shown that the optical properties of cholesteric materials can be accurately modeled with such a set of layers. This discretization then allows us to use the kMC protocol as described above to simulate the dynamics of circular depolarization. Yet, with the 30 layers, the parameter space becomes very large, and to reduce the number of computations needed, we simplify the system and consider thermally driven isomerization steps rather than photo‐induced molecular reorientations. By setting all twist angles between layers initially to −10° and the equilibrium angles *β*_eq_ all to +10°, we can still simulate helix inversion. To mimic the thermal isomerization, all interfaces are assigned the same probability for an isomerization event to happen. We just choose randomly at which interface in the stack an event occurs. Magnitude and direction of the change in twist angle are then decided as before, using Equation (4). Results for three different thermal isomerization runs are shown in Figure 6. As can be seen, the system indeed evolves to a state where the average twist angle is negative and close to *β*_eq_. Given a twist angle between all adjacent layers in the stack, we can calculate reflection and transmission of light. The degree of polarization in transmission *g*_trans_ during the thermal isomerization for a purely L‐polarized incident beam is shown in Figure 6B. At the start of the simulation, the stack is in a cholesteric state, and the transmitted light is still highly polarized (*g*_trans_ ~ 1.7). Then, with time, the average twist angle decreases and crosses zero. At first, the degree of polarization of the transmitted light slightly increases with *g*_trans_ reaching almost +2.0 for twist angles around 7°. Here, the absence of any depolarization indicates that the helical pitch length perfectly matches the wavelength of light. One the average twist angle goes below 7°, *g*_trans_ rapidly drops its helix inversion process. *g*_trans_ reaches a minimum of about 0.2 when the average twist angle crosses zero. Here, the stack is in a nematic‐like, transient state of the cholesteric helix inversion characterized by maximum depolarization of the incident circularly polarized light. Finally. The average twist angle slowly settles towards −7°, and the degree of polarization increases again towards ~1.9. The low depolarization in this final stretch of the simulation indicates that the pitch length again matches the wavelength of light. Overall, the simulation supports our previous assignment of the minimum in *g*_trans_ in Figure 1 to such a nematic transition state.

## 3 CONCLUSION

Polarization modulation spectroscopy in combination with a differential photon counting detection scheme provides a method to monitor the dynamical helical organization in films of polymer bearing photo‐isomerizable moieties. The differential photon counting, originally developed for circular polarization of luminescence spectroscopy, can also be used to monitor the photo‐imprinting of chirality through measurement of the depolarization of the circularly polarized light beam used in the imprinting process. Although such transmitted beams are usually quite intense, especially in comparison with fluorescence signal, we have shown here that despite the pulse pile‐up issue in counting individual photo pulses, a time resolution of 1 s with systematic and random errors on the order of a percent is feasible. Combined with the fact that the detection does not require external calibration, the differential photon counting seems the preferred method for direct monitoring of photo‐imprinting of chirality in real time by recording circular depolarization of the main beam.

The study of the photo‐imprinting also needs models and theory to interpret data. Optical models involving optical constants describing the collective response of a large number of molecules may provide a possible explanation for the photo‐imprinting based on the kinetic Monte Carlo simulations detailed here. We note that rates for isomerization process, for example, Equation (4), are still largely unknown and any chiral friction in the helix inversion process is currently at best only partly understood. The kinetic Monte Carlo method used here provides a convenient tool to test various expression for rates and effects of friction for compatibility with experimental data. Therefore, the simulation results presented here should be viewed as an effort to explore possible explanations for photo‐imprinting of chirality rather than a definitive account of its mechanism.

With regards to possible further development, we note that circular depolarization spectroscopy discussed here may be seen as a first step towards a more general circularly polarized dynamic light scattering as a future tool to study structure and dynamics of helical organization in materials and microstructures.

## Supporting information


Figure S1. Details on the circularly polarized irradiation beam.

## Data Availability

The data that support the findings of this study are available from the corresponding author upon reasonable request.
