# Supplementary material for: Circular depolarization spectroscopy: A new tool to study photo‐imprinting of chirality
Source: Chirality. 2023 Jan 13;35(3):147–54. doi: 10.1002/chir.23527 (PMC10108228; doi:10.1002/chir.23527)
Supplement: Supplementary file 1 — Figure S1. Details on the circularly polarized irradiation beam. [file CHIR-35-147-s001.pdf]

Supporting information

Circular depolarization spectroscopy : a new tool to study photo-imprinting of chirality.

Chidambar Kulkarni, Hirotoshi Sakaino, Ghislaine Vantomme, Stefan C.J. Meskers

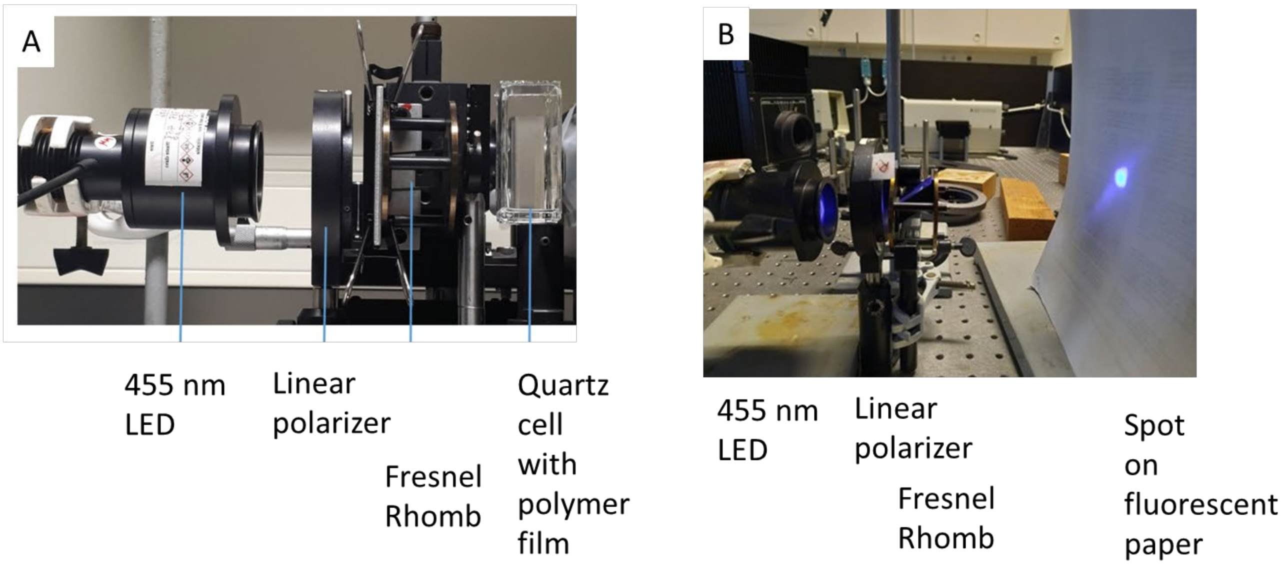

Figure S.1 A. Photograph of the LED light source and optical element to prepare the polarization of the illumination. Line up: mounted LED light source (M455L3 Thorlabs, USA) with adapter (COP1-A, with ACL50832U-A Aspheric Condenser Lens, Ø2", f=32 mm, NA=0.76, anti-reflection coating : 350-700 nm, B270 Optical Crown Glass, Thorlabs USA) followed by a linear polarizer (Oriel, 25010, Oriel, USA) and Frensel Rhomb (Ph. & F. Pellin, Paris, France, as used in ref. 29: P.H. Schippers and H. P. J. M. Dekkers Anal. Chem. 1981) B. Picture of the actual circularly polarized beam.
